# Sexualaufklärung für Jugendliche und Erwachsene – Max Hodann (1894–1946) in Aktion

**DOI:** 10.1007/s00120-024-02443-y

**Published:** 2024-09-27

**Authors:** Florian Mildenberger, Nils Hansson, Göran Bergkvist, Friedrich H. Moll

**Affiliations:** 1 Institut für Geschichte, Theorie und Ethik der Medizin, Centre for Health and Society, Medizinische Fakultät, Heinrich-Heine-Universität, Düsseldorf, Deutschland; 2 Urologischer Arbeitsplatz Krankenhaus Merheim, Urologische Klinik, Kliniken der Stadt Köln GmbH, Neufelder Straße 32, 51067 Köln, Deutschland; 3 Deutsche Gesellschaft für Urologie, Düsseldorf-Berlin, Deutschland; 4 Abteilung für Geschichte der Medizin, Universität Lund, Lund, Schweden

**Keywords:** Max Hodann, Sexualaufklärung, Sexualität, Geschlechtskrankheiten, Medizin im Ostseeraum, Max Hodann, Sex education, Sexuality, Sexually transmitted diseases, Medicine in the Baltic Sea region

## Abstract

Bis in die 1970er-Jahre hinein tobte in Deutschland ein Kulturkampf über die Frage der Sinnhaftigkeit einer sexuellen Aufklärung der Jugend. Doch ein Arzt erkannte frühzeitig, dass es mehr Sinn machen würde, auch die Erwachsenen über ihren Körper und seine genitalen Leiden aufzuklären. Damit spülte Max Hodann (1894–1946) während der Weimarer Republik ungewollt zahllose Patienten in die Praxen der „neuen Fachärzte“.

## Abstract (en)

Up to the 1970s, a cultural battle raged in Germany and Europe about the question of the sense to inform and educate young people about gender, sex, and sexuality. One physician realized early that it is important to educate adults about their bodies and their genital and genitourinary disorders. Max Hodann (1894–1946), thus, unintentionally flooded urological practices with countless patients.

## Max Julius Carl Alexander Hodann: eine kurze biografische Notiz

Bis in die 1970er-Jahre hinein tobte in Deutschland ein Kulturkampf über die Frage der Sinnhaftigkeit einer sexuellen Aufklärung der Jugend. Doch ein Arzt erkannte frühzeitig, dass es mehr Sinn machen würde, auch die Erwachsenen über ihren Körper und seine genitalen Leiden aufzuklären. Damit spülte Max Hodann (1894–1946) ungewollt zahllose Patienten in urologische Praxen.

**Abb. 1:**
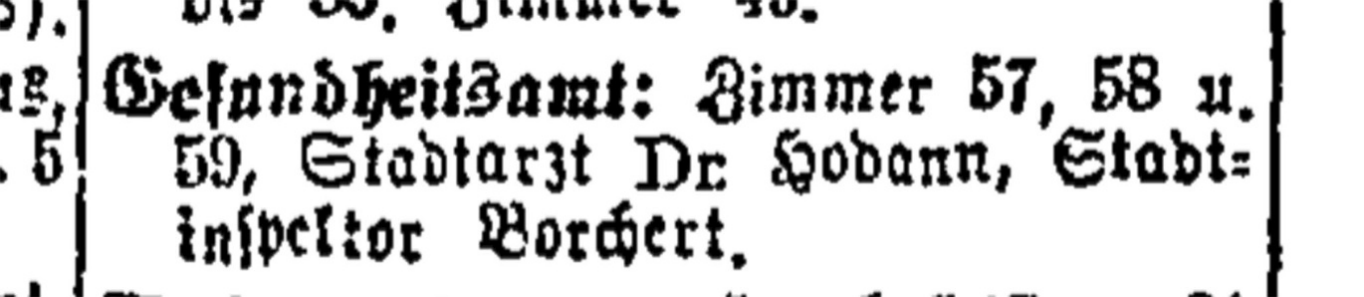
Ausriss Berliner Adressbuch 1925, Scherl-Verlag, Teil III, Behörden, Kirchen, öffentliche Einrichtungen und Schulen in Groß-Berlin S. 122

Geboren als Sohn des Oberstabsarztes Carl Hodann im oberschlesischen Neiße (poln. Nysa), wuchs der junge Max zunächst in Meran heran, ehe er ab 1903 das humanistische Gymnasium in Berlin-Friedenau besuchte. Hier kam er früh mit der elitären Philosophie von Leonard Nelson (1882–1927; [1, 2]) in Berührung [3]. Durch den Fronteinsatz im Ersten Weltkrieg desillusioniert, begann sich Hodann während seines Medizinstudiums in Berlin Fragen der Sozialhygiene zuzuwenden. Er hörte Vorlesungen bei dem ersten deutschen Lehrstuhlinhaber in Berlin und wichtigen Protagonisten der Sozialhygiene Alfred Grotjahn (1869–1931; [4]), der auch Hodanns Dissertation über die „sozialhygienische Bedeutung der Beratungsstellen für Geschlechtskranke“ betreute [5, 6]. Hierin benannte Hodann die drei Säulen einer erfolgreichen Arbeit: Anonymität der Daten, Gleichbehandlung von Männern und Frauen und umfängliche Sexualaufklärung. Letztere avancierte alsbald zum Lebenswerk des jungen Arztes [7]. In niedergelassener Praxis und als Leiter des Gesundheitsamtes in Berlin-Reinickendorf (ab 1923; Abb. 1) engagierte er sich für Mütterberatung und förderte die aus Österreich kommende pädagogische Bewegung der „Kinderfreunde“ [8].

Bereits 1921 war Hodann in die Gründung der ersten deutschen Kinderfreundegruppe in Nowawes bei Potsdam eingebunden [9]. Die Erziehung sollte gänzlich frei von religiösen Einflüssen erfolgen. Seine Hauptzielgruppen aber waren Jugendliche (ab 14 Jahre) und Erwachsene.

Hodann beschränkte sich nicht – wie die Mitglieder der bereits vom kaiserlichen Obrigkeitsstaat autorisierten im Jahre 1902 gegründeten „Deutschen Gesellschaft zur Bekämpfung der Geschlechtskrankheiten“ (DGBG), der u. a. der Urologe Carl Posner (1854–1928) oder Hodanns wissenschaftlicher Lehrer Alfred Grotjahn angehörten oder ärztlicher Standesvereine – auf Vorträge oder die Veröffentlichung kleiner Broschüren, sondern suchte die Angehörigen der Unterschichten direkt in ihrem Milieu auf [10]. Hodanns Erfolg gründete sich darauf, dass er den sexuellen Genuss und die Freude am geschlechtlichen Miteinander in den Mittelpunkt stellte, anstatt moralinsauer vor Geschlechtskrankheiten oder Sittenlosigkeit zu warnen [11]. Gekonnt zitierte er die Worte von Patientinnen aus seiner Sprechstunde, z. B. antwortete eine Frau auf die Frage, wie sie den Sex empfunden habe

*„Ich hab ja gar nicht gar nicht recht gewusst, was da vorgeht. Ach, ich weiß ja heute kaum Bescheid*“ [12].

Er gab Lehrerinnen, die Sexualkunde unterrichten sollten^1^, an der Aufgabe, selbst einen Fragebogen über sexuelle Grundkenntnisse auszufüllen, bereits scheiterten [13, S. 24]. Die Schüler wiederum lauschten begeistert seinen Aufklärungsvorträgen, wie sich noch Jahrzehnte später Zeitzeugen erinnerten [14]. Die Schülerin Eva Katter (1910–1995) wurde sich durch Hodanns Erklärungen ihrer eigenen Transsexualität bewusst und verwandelte sich sukzessive in Gerd Katter [15]. Der Evangelische Elternbund sorgte bereits 1925 dafür, dass Hodann in preußischen Schulen keine Vorträge mehr halten durfte [16] – infolgedessen konnten die Jugendlichen in ihrer Freizeit ohne Wissen von Pädagogen und Eltern die Aufklärungsveranstaltungen Hodanns besuchen. Die Sozialistische Arbeiterjugend sowie die Naturfreunde luden ihn in Volkshochschulen ein, wo er neben den Jugendlichen vor allem Erwachsene erreichte [17]. Stets an seiner Seite wirkte die Ehefrau Maria (1897–1976), die er 1919 geheiratet hatte und von der sich 1926 zwar scheiden ließ, aber weiter mit ihr zusammenarbeitete. Sie setzte sich insbesondere für eine stärkere Beteiligung von Frauen in der Sexualreformbewegung ein [18]. Gemeinsam mit ihr engagierte er sich auch für eine Liberalisierung des Abtreibungsstrafrechts [19]. So kam Hodann zügig in Kontakt zum Doyen der deutschen Sexualforschung, dem Arzt Magnus Hirschfeld (1868–1935), der seit 1919 ein eigenes Forschungsinstitut in Berlin aufbaute, das „Institut für Sexualwissenschaft“.

## Sexualforschung bei Hirschfeld und internationale Kontakte

Spätestens ab dem Jahr 1926 war Hodann fester Bestandteil von Hirschfelds Bemühungen und arbeitete am Institut in Berlin mit [20].

Max Hodann war mit dem der KPD nahestehenden Richard Linsert (1899–1933) im Jahre 1930 bei der Gründung eines “Archiv für Sexualwissenschaft”, aktiv, in dem auch Berndt Götz (1891– ) sowie der Jurist Fritz Flato (1891–1949 New York) mitwirkten, da es zwischen den Mitarbeitern Hirschfelds zu dieser Zeit Auseinandersetzungen mit Hirschfeld bei gemeinsamen auflagenstarken Buchpublikationen um Honorare gab, bei deren Diskussion auch Hodann aus dem Institut ausschied [21, 22].

**Abb. 2:**
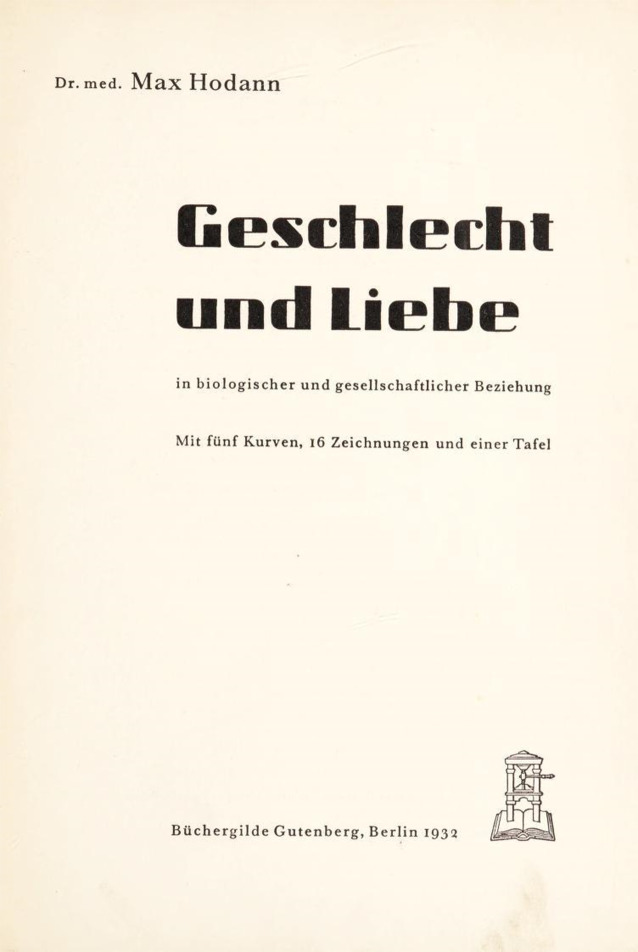
Frontispiz Max Hodann Geschlecht und Liebe 1931. (Sammlung Moll, Repro Moll-Keyn, mit freundl. Genehmigung)

**Abb. 3:**
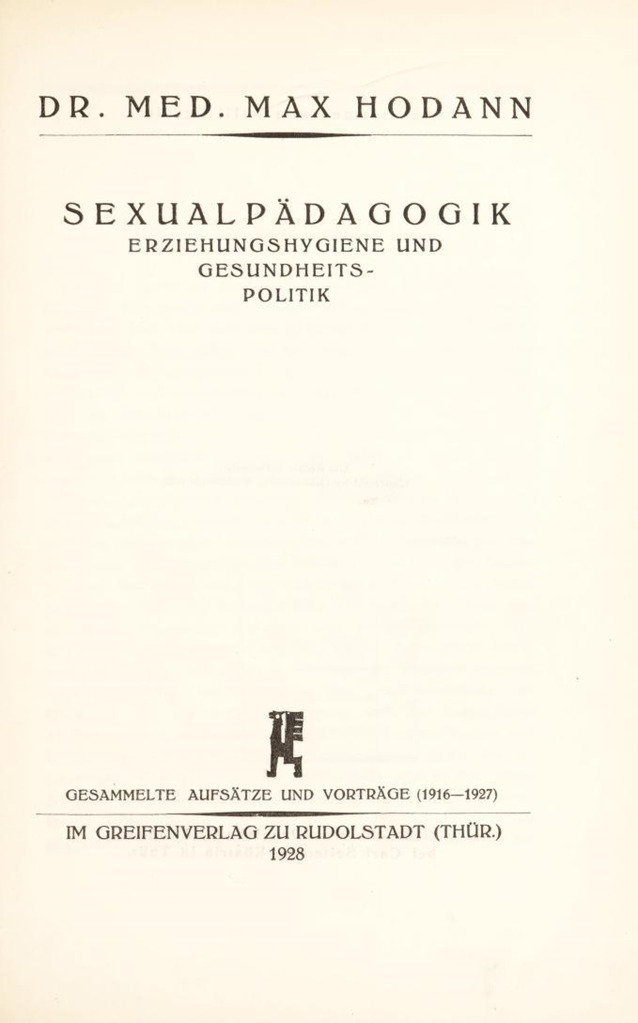
Max Hodann Sexualpädagogik 1928. (Sammlung Moll, Repro Moll-Keyn, mit freundl. Genehmigung)

In den folgenden Jahren sollte er bei Fragen der heterosexuellen Sexualaufklärung seinen Lehrmeister sogar an Relevanz überflügeln. Hierzu trugen entscheidend seine vielfach aufgelegten Bücher bei: „Bringt uns wirklich der Klapperstorch?“ [23], „Bub und Mädel“, [24] „Die Sexualnot der Erwachsenen“, [25] „Geschlecht und Liebe“, „Elternhygiene“, „Sexualpädagogik“ sowie „Sexualelend und Sexualberatung“ [26]. Das mehrte auch die Zahl seiner Gegner. (Abb. 2, 3, 4 und 5).

**Abb. 4:**
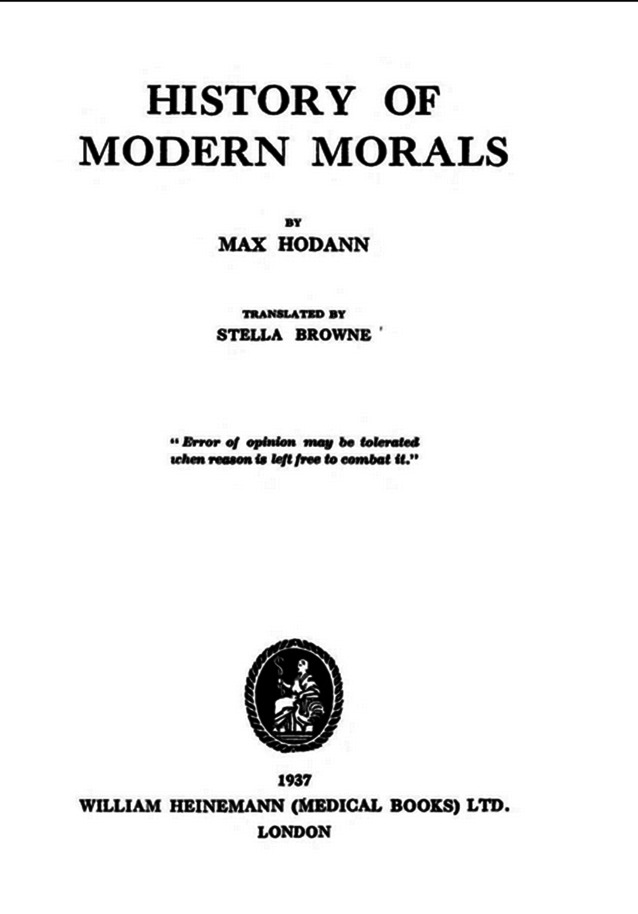
Frontispiz der englischen Ausgabe Geschichte der modernen Moral, 1937. (Repro Moll-Keyn, mit freundl. Genehmigung)

**Abb. 5:**
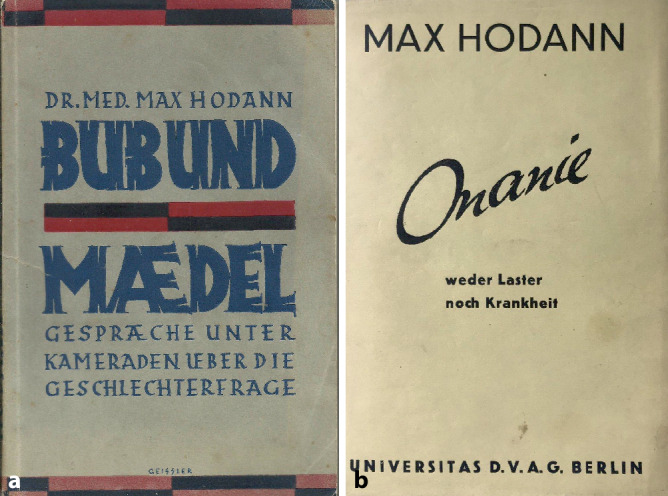
**a** Buchcover Max Hodann Bub und Mädel, Greifen-Verlag 1929 vorherige Auflagen Oldenburg-Verlag, **b** Onanie weder Laster noch Krankheit 1931. (Sammlung Moll, Repro Moll-Keyn, mit freundl. Genehmigung)

Die Tätigkeit als erster Vorsitzender des „Bundes der Freunde der Sowjetunion“ ab 1928 und der Arbeit in der „Internationalen Arbeiterhilfe“ trugen ebenfalls nicht dazu bei, Hodanns Ansehen bei konservativen Ärztevertretern zu verbessern [27]. 1928 wurde Hodann wegen angeblicher „Sittenlosigkeit“ seiner Aufklärungsbücher angezeigt, aber freigesprochen [28]. Das Verfahren richtete sich auch gegen Hodanns Veröffentlichungsort, den Greifen-Verlag in Rudolstadt [29]. Insbesondere die katholische Moraltheologie und Hodann gerieten zunehmend aneinander. Hodann unterstellte den Gegnern, „theologische Voreingenommenheit“, die sie daran hindere, sich überhaupt mit den Problemen von Jugendlichen und Erwachsenen auseinanderzusetzen [30]. Der Jesuit Joseph Schröteler (1886–1955) bezeichnete Hodann erstmals 1929 als entscheidender Mitverursacher für das Anwachsen von Nacktkultur, Bubikopf und die lockere Haltung zum vorehelichen Geschlechtsverkehr in Deutschland [31]. Bereits in der Sprache zeigten sich die Unterschiede zwischen den Theologen und Hodann, erstere schrieben konsequent von „Zöglingen“, Hodann von „Menschen“. Er revanchierte sich mit einem Vortrag auf dem Kongress der Weltliga für Sexualreform in Kopenhagen und benannte seinerseits die katholischen Theologen als seine Hauptgegner [32]. Bisweilen nannte Hodann die katholischen Theologen „Prediger des Todes“ [33]. Hauptstreitpunkt war die von Hodann avisierte Ungefährlichkeit der Masturbation, die aus Sicht der protestantischen und katholischen Kleriker den Einstieg in die Entwicklung eines individuellen Körperbewusstseins darstellte und so die Gefahr einer Abkehr von der religiösen Gemeinschaft implizierte [1]. Die Theologen konzentrierten sich voll und ganz auf Hodanns Jugendaufklärungsbücher und übersahen die Wirkmächtigkeit seiner Veröffentlichungen über das Sexualleben der Erwachsenen. Denn Hodann informierte durch Vorträge und preiswerte Publikationen eine ganze Generation von jungen Erwachsenen über die Befindlichkeiten, Probleme und Genussmöglichkeiten des eigenen Körpers. Er empfahl stets, Ärzte zu konsultieren, doch öffentliche Gesundheitsämter garantierten keine Anonymität der Daten. Infolgedessen profitierte eine kleine Gruppe von Ärzten in hohem Maße von Hodanns Engagement. Urologen waren bislang der verlängerten Arm von Kliniken gewesen, aber der Ansturm der rudimentär aufgeklärten jungen Männer und Frauen aus allen gesellschaftlichen Schichten veränderte in den 1920er-Jahren das Fach besonders im Großstadtbereich.

Zum einen waren auf dem Bremer Ärztetag 1924 die einzelnen Facharztgruppen festgelegt worden [34]. Hierbei waren für das allgemeine Publikum sowohl Urologen wie Dermatologen ersichtlich, wobei die Dermatologie häufig mit der Venerologie verbunden wurde, was immer eine bestimmte Stigmatisierungsgefahr beim Praxisbesuch von Patienten bedeutete. Der Besuch beim Urologen, dem früheren „Spezialarzt für Erkrankungen der Harnorgane“, war hingegen immer unauffälliger. Auch verfügten gerade im Kurbereich Urologen über eine große Therapiebreite. Exemplarisch waren hier die Lehrbücher des Münchner Felix Schlagintweit (1888–1950; Kurarzt in Bad Brückenau) „Urologie des Praktischen Arztes“ oder des Kölner Berthold Goldberg (1866–1925; Kurarzt in Bad Wildungen) „Kleine Urologie“. In ihnen schließt sich die konsekutive Harnröhrenstrikturtherapie an die Kapitel der Infekttherapie direkt an, um neben der medikamentösen Behandlung die instrumentelle, transurethrale Therapie als weiteren wichtigen Therapiebaustein, den in der Regel nur ein Urologe in hoher Kunstfertigkeit und mit entsprechendem Instrumentarium der Oberländerschen oder Kollmannschen Bougies virtous beherrschte, herauszustellen[35–40].

**Abb. 6:**
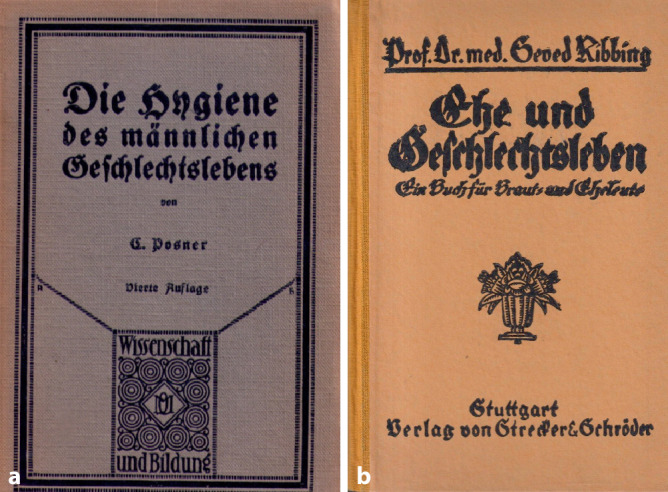
**a** Carl Posner (1854–1928) „Die Hygiene des männlichen Geschlechtslebens, Frontcover, 3. Auflage 1918, Quelle und Meyer, Leipzig, **b** Seved Ribbing (1845–1921) „Ehe und Geschlechtsleben“ Frontcover, hier in der Auflage von 1927, Strecker und Schröder, Stuttgart. (Sammlung Moll, Repro Moll-Keyn, mit freundl. Genehmigung)

Bereits der Urologe und AeGeSe (Ärztliche Gesellschaft für Sexualmedizin und Eugenik) Mitglied Carl Posner (1854–1928), der ebenfalls ein mehrfach aufgelegtes Werk „Therapie der Harnkrankheiten“ [41] publiziert hatte, in welchem das entsprechende Gebiet vollumfänglich dargestellt worden war, hatte in seiner „Hygiene des männlichen Geschlechtslebens“ [42], auf die Bedeutung einer spezialärztlichen Behandlung hingewiesen [43]. Auch in weiteren Ratgebern wie dem des eher konservativen Schweden Seved Ribbing (1845–1921), Lund, der in Deutschland über mehr als 40 Jahre bis in die NS-Zeit in nur geringen Bearbeitungen erschien, wurde auf die Bedeutung der speziellen ärztlichen Behandlung hingewiesen^2^ ([44]; Abb. 6).

**Abb. 7:**
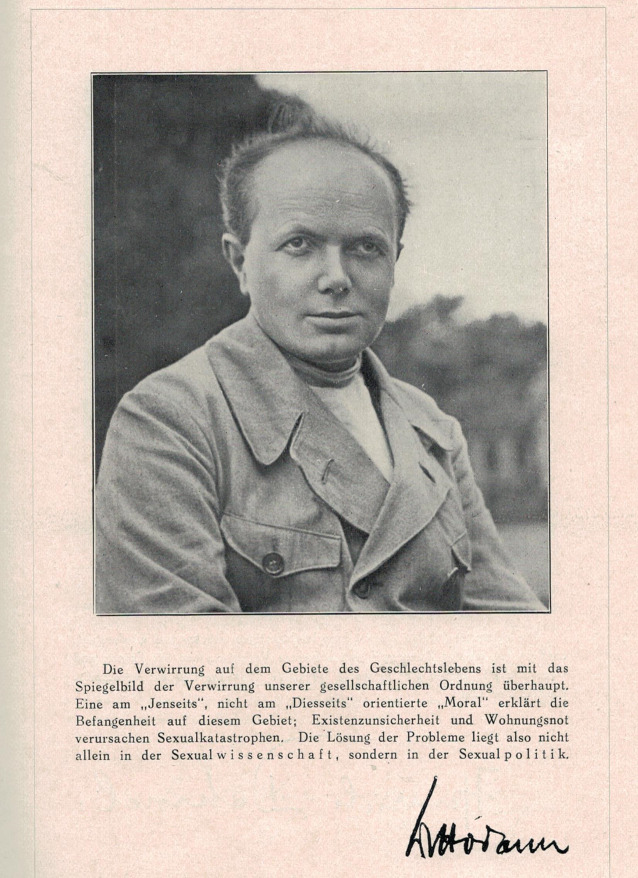
Max Hodann (1894–1946) aus Kaiser M 1928 Liebeslehre, Kultur-Verlag. (Repro Moll-Keyn, mit freundl. Genehmigung, S VII)

Die Feindschaft der Theologen blieb Hodann erhalten. Sein Auftritt beim letzten Kongress der Weltliga für Sexualreform 1932 im tschechischen Brünn wurde als Ausdruck „antireligiöser Einstellung“ in der Zeitschrift *Caritas* interpretiert [45]. Längst war er zum Hauptgegner jeder christlichen Sexualpädagogik avanciert [46, 47]. Die Nationalsozialisten erkannten in Hodann den Sozialisten und somit einen politischen Feind [48]. Sein sexualreformerisches Oeuvre hingegen ließen sie weitgehend unangetastet, da es sich nicht sonderlich von den eigenen Konzeptionen unterschied. Auch die Nazis wollten den Einfluss der Kirchen zurückdrängen, die heterosexuellen Jugendlichen sexuelle Freiheiten genießen lassen und dies mit Gesundheitsaufklärung verbinden [49]. Auch hinsichtlich der Eugenik gab es Überschneidungen. Hodanns Werke wurden zwar ebenso wie die Hirschfelds verbrannt, waren aber dennoch (nicht alle) weiterhin lieferbar. Hodann selbst wurde umgehend inhaftiert, in „Schutzhaft“ verbracht und erst im November 1933 entlassen. Umgehend verließ er Deutschland. Nach dem Tod Hirschfelds 1935 im Exil schrieb Hodann den weithin beachteten Nachruf im *Internationalen Ärztlichen Bulletin* [50]. Er selbst befand sich nach einer Odyssee durch die Schweiz 1933, Palästina, die Niederlande, Norwegen, Frankreich und Dänemark zu dieser Zeit in Großbritannien und plante die Zusammenführung der geretteten Bestände sexualreformerischer Bibliotheken aus Deutschland und den Aufbau eines neuen „Instituts für Sexualwissenschaft“ [51]. Dieser Plan zerschlug sich, weil die britischen Behörden ihn als Kommunisten identifizierten und das Visum nicht verlängerten. Ende 1935 musste Hodann England verlassen. Der beständige Stress führte zum Ausbruch einer Asthmaerkrankung, woraufhin die UdSSR ihm die Einreise verweigerte. Er floh nach Skandinavien, wo er wieder eine rege Publikations- und Vortragstätigkeit entfaltete (Abb. 7).

## Hodann in Schweden

In Schweden fand Hodann Unterschlupf bei Elise Ottesen-Jensen (1886–1973) in Stockholm [52–55]. Durch ihre Arbeit innerhalb der World League for Sexual Reform (WLSR) hatte Ottesen-Jensen Hodann und den Kopenhagener Arzt Joyce Leeunbach (1884–1955) kennengelernt – beide wohnten während ihres Flüchtlingsaufenthalts in Stockholm als Archivarbeiter bei „Riksförbundet för sexuell upplysning“ (RFSU [Reichsverbund für sexuelle Aufklärung]; John Ericssonsgatan 6).

Sie motivierte die Leitung des „Riksförbundet för sexuell upplysning“ (RFSU), sich für Hodanns Aufenthaltserlaubnis einzusetzen [56]. In ihrer Autobiografie charakterisiert sie ihn als

„…[e]ine kraftvolle Persönlichkeit, Organisator, Redner und Autor. Sehr radikal und jung. Seine

Sexualaufklärungsbücher wurden nicht nur unter deutschen Jugendlichen weit verbreitet

aber auch in mehreren anderen Ländern, auch in Schweden.“

**Abb. 8:**
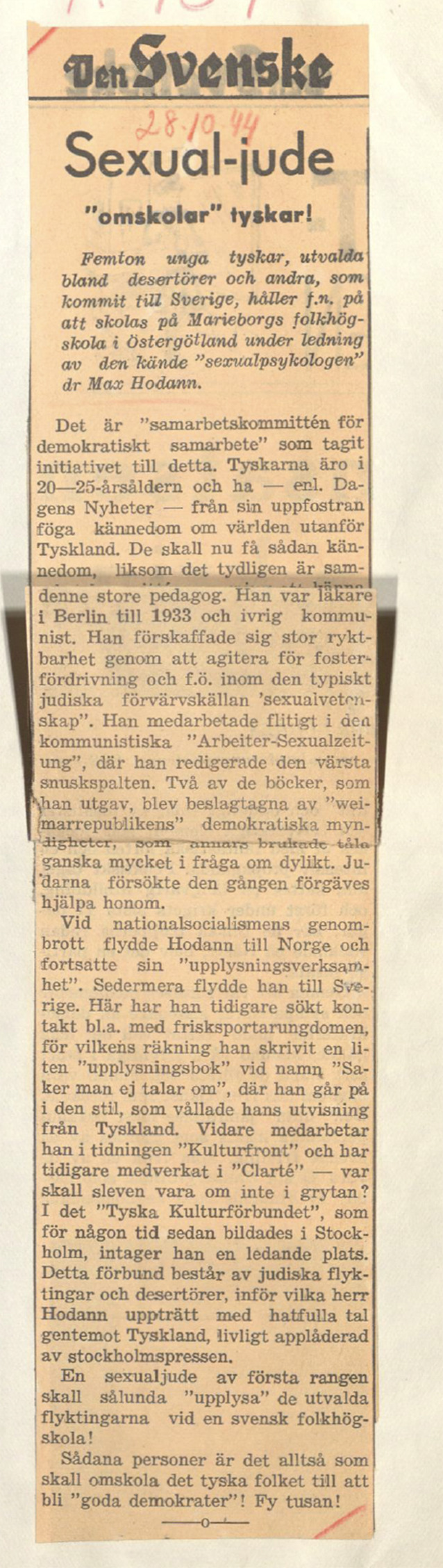
Bildtext: Ingress, übersetzt aus dem Schwedischen „Fünfzehn junge Deutsche, ausgewählt aus Deserteuren und anderen, die nach Schweden gekommen sind, besuchen derzeit unter der Leitung des bekannten ‚Sexualpsychologen‘ Max Hodann die Volkshochschule Marieborg in Östergötland“. […]. Der Lehrer dieser hübschen Jungen ist – der Jude Max Hodan! […] Im seit einiger Zeit gebildeten „Deutschen Kulturbund“ in Stockholm nimmt er einen Spitzenplatz ein. Dieser Bund besteht aus jüdischen Flüchtlingen und Deserteuren, vor denen Herr Hodann hasserfüllte Reden hielt gegen Deutschland, begeistert von der Stockholmer Presse gefeiert.“ Den svenske folksocialisten 28.10.1944

Nach kurzer Zeit sah sich Hodann wieder mit Vorwürfen konfrontiert, er sei ein „Sexual-Jude“ ([57, 58]; Abb. 8).

Diese wurden vom schwedisch-nationalsozialistischen Studentenverein geschürt. Gleichwohl führte er seine Arbeit weiter, beriet Eheleute bei sexuellen Problemen und durfte schließlich im Herbst 1944 die sexuelle Reedukation von jungen deutschen Deserteuren begleiten, was die schwedische Presse aufgriff [57, 59]. Darüber hinaus engagierte er sich in pazifistischen Kreisen in Stockholm. In seiner Akte der Schwedischen Sicherheitspolizei wird erläutert, dass Hodann an einem Treffen am 26. Oktober 1943 in Stockholm teilgenommen habe, organisiert vom schwedischen Schriftsteller und Brecht-Übersetzer Arnold Ljungdahl (1901–1968; [60]). Dabei signierten Hodann und 22 weitere deutsche Migranten aus Wissenschaft, Kultur und Politik, darunter der Bildhauer Karl Helbig (1897–1951), der Schauspieler Curt Trepte (1902–1990) und der Komponist Leo Blech (1871–1958) einen offenen Brief an Erich Weinert (1890–1953), Robert Kuczynski (1876–1947) und Thomas Mann (1875–1955). In dem Schreiben plädierten sie für ein „neues freies Deutschland“ und die Bekämpfung des Nationalsozialismus. Die überregionalen Zeitungen *Aftonbladet* und *Aftontidningen* berichteten darüber, was vermutlich zum Interesse der Sicherheitspolizei beitrug.

Doch nach dem Kriegsende verschlechterte sich sein Gesundheitszustand erneut und schließlich starb Max Hodann am 17. Dezember 1946 infolge eines Asthmaanfalls.

Sein Ruhm verblasste jedoch auch nicht nach seinem Tod. In der DDR wurden seine Werke im nicht verstaatlichten Greifen-Verlag, Rudolstadt bis in die 1970er-Jahre unverändert nachgedruckt. Dies lässt Rückschlüsse darauf zu, wie prekär die Lage der Arbeiterschaft und ihrer medizinischen Versorgung und Gesundheitsaufklärung im real existierenden Sozialismus blieb, sobald es sich um den Bereich unterhalb der Gürtellinie handelte. Hodann blieb so auch nach seinem Tode für parteigebundene Sozialisten ein Ärgernis. In Westberlin bewarb der FKK-Prophet Adolf Koch (1897–1970) weiterhin Hodanns Erbe. In der Bundesrepublik jedoch schien er in Vergessenheit zu geraten, was die katholischen Moraltheologen sicher erfreute. Die Phase der Nemesis änderte sich abrupt im Sommer 1969, als die Boulevardpresse Wind von den Anstrengungen der sozialdemokratischen Jugendorganisation „Die Falken“ zur Entfesselung einer sexuellen Revolution beim eigenen Nachwuchs bekam. Bereits bei einem Zeltlager in den Vogesen 1968 war mit antiautoritärer Pädagogik experimentiert worden [61]. Im Juni 1969 wurde im schwedischen Norrahammar direkt an die Traditionen der Kinderfreunde aus den 1920er-Jahren angeknüpft. Dabei spielte die Sexualerziehung und Befreiung der Jugendlichen von Tabus und Vorurteilen eine zentrale Rolle. Aufgeschreckt durch Berichte in der Presse, meldete sich der Berliner Senator für Familie, Jugend und Sport, Horst Körber (1927–1981) bei der Bundesleitung der „Falken“ und verlangte Aufklärung. Der Landesverband Berlin ließ den Senator wissen, man bewege sich auf den Pfaden der Sexualreform der 1920er-Jahre und verwies u. a. auf die Schriften Hodanns, die in der SPD offenbar mittlerweile völlig vergessen worden waren^3^. Der selbst berufene Sexualaufklärer Ernest Borneman (1915–1995) kürte sich selbst zum Nachfolger und Erben Max Hodanns [62]. Es folgte eine langsame Wiederentdeckung durch Sexualwissenschaftler und insbesondere die Mitarbeiter der Magnus-Hirschfeld-Gesellschaft im Laufe der 1980er-Jahre. Jedoch gilt Hodann bis heute als „Aufklärer der Jugend“, seine zeitgenössische Wirkmächtigkeit für Erwachsene spielt keine Rolle. Seine Relevanz für die Weiterentwicklung des ärztlichen Standes stellt ebenfalls ein Forschungsdesiderat dar.

## Fazit für die Praxis

- Wir haben in dieser Studie versucht anhand des Umfeldes, in dem sich Urologen und Venerologen in der Weimarer Republik bewegten, die Bezüge aufzuzeigen, die das Fachgebiet einem weiteren Publikum vertraut machten. Gerade hier boten sich besondere Möglichkeiten für niedergelassene Urologen oder Kurärzte mit urologischem Fokus, durch das Erstarken der Sexualmedizin und -beratung in breiten Gesellschaftskreisen jenseits des Mittelstandes neue Patientenschichten zu erreichen.
- Der Schwerpunkt erschließt sich gerade nicht in den rein wissenschaftlichen Publikationen wie der *Zeitschrift für Urologie* (Georg Thieme, Leipzig) oder der *Zeitschrift für Urologische Chirurgie* (Julius Springer, Berlin). Diese wandten sich in der Regel an Krankenhausärzte.
- Analysen im Sinne einer Versorgungsforschung sollten erst im 21. Jahrhundert Einzug in die Fachpresse oder die Gremien der 1906 gegründeten Fachgesellschaft halten.

## Footnotes

1 Das Fach Sexualkunde war in Preußen formal bereits im Jahre 1900 (2. September) eingeführt worden. Vgl. [13, S. 23–29].

2 Eine Publikation zu Seved Ribbing ist in Vorbereitung.

3 Archiv zur Geschichte der Arbeiterjugendbewegung Oer-Erkenschwick: SJD-MR-AC 19/13, Brief der Falken an Körber vom 29.08.1969 Noch immer gibt es keine weiterführenden Eintrag in der Deutschen Biographie zu seinem Namen.
